# Euglycemic Diabetic Ketoacidosis With Sepsis and Acute Kidney Injury in Critically Ill Patients: A Case Series

**DOI:** 10.7759/cureus.83920

**Published:** 2025-05-11

**Authors:** Daichi Yomogida, Suguru Hasegawa, Shiori Mizuta, Shinjiro Horikawa, Yosinao Koshida

**Affiliations:** 1 Department of Intensive Care Medicine, Toyama Prefectural Central Hospital, Toyama, JPN

**Keywords:** acute kidney injury, anion gap, continuous hemodiafiltration, euglycemic diabetic ketoacidosis, kidney replacement therapy

## Abstract

Diabetic ketoacidosis (DKA) typically presents with hyperglycemia, but euglycemic diabetic ketoacidosis (EDKA) occurs when patients present with acidosis and normal blood glucose levels. This condition has garnered attention due to its association with sodium-glucose cotransporter-2 (SGLT2) inhibitors and other factors such as sepsis. Acute kidney injury (AKI) often complicates sepsis cases and is associated with metabolic acidosis. Distinguishing between AKI and EDKA can be difficult, particularly when both conditions co-occur, as observed in three ICU cases of sepsis with AKI and EDKA. In this report, the clinical challenges of diagnosing EDKA in critically ill patients, especially in the presence of metabolic acidosis and an elevated anion gap, are highlighted. In these cases, metabolic acidosis was initially attributed to AKI; however, blood ketone levels were essential for confirming EDKA. Treatment for EDKA was prioritized over kidney replacement therapy (KRT), which was only initiated when needed for AKI management. Despite its potential benefits in managing AKI, KRT's role in EDKA treatment requires further investigation. This report emphasizes the importance of early diagnosis of EDKA in diabetic patients with sepsis and AKI, stressing the need for prompt ketone testing and insulin therapy, while acknowledging the ongoing uncertainties surrounding the impact of KRT on prognosis in EDKA cases.

## Introduction

Diabetic ketoacidosis (DKA) is caused by an absolute or relative deficiency of insulin and is characterized by hyperglycemia, thereby constituting one of the hyperglycemic crises. However, a subset of patients with DKA present with normal blood glucose levels in the presence of acidosis - a condition referred to as euglycemic diabetic ketoacidosis (EDKA) [1]. In recent years, the significance of EDKA has been re‐emphasized following multiple reports of cases induced by sodium-glucose cotransporter-2 (SGLT2) inhibitors. EDKA is a life-threatening diabetic complication that can be fatal, and delays in its diagnosis or therapeutic intervention may worsen patient outcomes. Nonetheless, it has long been recognized that various mechanisms, including sepsis, can contribute to its development [2].

 Acute kidney injury (AKI) due to sepsis is a relatively common organ dysfunction in sepsis, affecting 10-67% of patients with sepsis [3]. In cases of renal dysfunction, including AKI, metabolic acidosis with an elevated anion gap (AG) is often observed. When both EDKA and AKI are present in sepsis, metabolic acidosis with an elevated AG complicates the differential diagnosis of the underlying pathology.

In this study, we present three cases of sepsis complicated by both AKI and EDKA, and we discuss their clinical characteristics and the challenges in distinguishing between the two conditions.

## Case presentation

Case 1

A 66-year-old man with a history of a remote myocardial infarction presented to our hospital with abdominal discomfort secondary to urinary retention. His medications at admission included insulin degludec and insulin aspart for type 2 diabetes mellitus.

On physical examination, his blood pressure was 92/65 mmHg with warm extremities, heart rate was 88 beats/min, and oxygen saturation was 95% on room air. Abdominal examination revealed distention in the lower abdomen.

Laboratory studies showed a leukocyte count of 25,500/μL, a creatinine level of 10.45 mg/dL, and a C-reactive protein level of 27.95 mg/dL. Arterial blood gas analysis demonstrated a pH of 7.247, pCO₂ of 18.3 mmHg, pO₂ of 101.0 mmHg, HCO₃⁻ of 7.7 mmol/L, and lactate of 0.9 mmol/L (Table 1).

**Table 1:** Laboratory data on admission The ICU admission laboratory findings of the subjects are presented. In all cases, blood glucose levels were normal to slightly elevated. Although all patients exhibited metabolic acidosis with an elevated AG, with the exception of case 3, who presented with septic shock, lactate levels remained within the normal range. AcAc, acetoacetate; AG, anion gap; Lac, lactate; 3-OHBA, 3-hydroxybutyrate

	Na (mEq/L) (ref range: 136–143 mEq/L)	Cl (mEq/L) (ref range: 98–108 mEq/L)	pH (7.35–7.45)	HCO_3_^-^ (mmol/L) (ref range: 22–28 mmol/L)	Lac (mmol/L) (ref range: 1–1.5 mmol/L)	Adjusted AG (ref range: 10–14)	Blood glucose (mg/dL) (ref range: 80–180 mg/dL)	Creatinine (mg/dL) (ref range: 0.65–1.07 mg/dL)	Ketone bodies (μmol/L) (ref range: 0–131 μmol/L)	AcAc (μmol/L) (ref range: 0–55 μmol/L)	3-OHBA (μmol/L) (ref range: 0–85 μmol/L)	Ketone bodies in urine
Case 1	129	91	7.247	7.7	0.9	33.6	184	10.45	5038	1526	3512	N/A
Case 2	148	113	7.345	12.8	1.2	26.45	134	2.94	10312	3064	7248	N/A
Case 3	136	105	7.316	14.7	2.2	19.55	103	2.05	N/A	N/A	N/A	Positive

Urinary retention was confirmed, and drainage was achieved with an indwelling urethral catheter.

The patient was diagnosed with sepsis secondary to a urinary tract infection and AKI and admitted to the intensive care unit (ICU). Although the elevated corrected anion gap suggested the accumulation of non-volatile acids, low lactate levels prompted measurement of blood ketones to evaluate for ketoacidosis. With persistent oliguria and worsening metabolic acidosis, continuous hemofiltration dialysis (CHDF) was initiated. Urine output subsequently recovered and metabolic acidosis improved, allowing CHDF to be discontinued on hospital day 3. The patient was discharged from the ICU on hospital day 6.

Case 2

A 78-year-old man with a history of pituitary adenoma was admitted with multiple rib fractures. His medications at admission included dapagliflozin for type 2 diabetes mellitus. He developed aspiration pneumonia and, owing to a rapid deterioration in respiratory status, was transferred to the ICU.

On examination, his blood pressure was 100/60 mmHg, heart rate was 90 beats/min, and oxygen saturation was 88% with a reservoir mask.

Laboratory evaluation revealed a leukocyte count of 12,400/μL, a creatinine level of 1.7 mg/dL, and a C-reactive protein level of 2.94 mg/dL. Arterial blood gas analysis showed a pH of 7.345, pCO₂ of 32.8 mmHg, pO₂ of 64.6 mmHg, HCO₃⁻ of 12.8 mmol/L, and lactate of 1.2 mmol/L (Table 1).

The patient was intubated, and CHDF was performed from hospital day 2 to day 4 for an acute exacerbation of chronic renal failure. Metabolic acidosis with elevated anion gap was also noted in this patient, and we performed the measurement of blood ketones. Due to difficulties in clearing secretions, extubation was not achieved, and the patient was discharged from the ICU on hospital day 12 following tracheostomy.

Case 3

An 84-year-old man with a history of laparoscopic sigmoid colon resection for rectal carcinoma eight days prior developed septic shock secondary to peritonitis and was admitted to the ICU. His medications at admission included pioglitazone and semaglutide for type 2 diabetes mellitus.

On examination, his blood pressure was 90/45 mmHg, heart rate was 130 beats/min, and oxygen saturation was 98% on 40% fraction of inspired oxygen.

Laboratory studies revealed a leukocyte count of 12,100/μL, a creatinine level of 2.05 mg/dL, and a C-reactive protein level of 17.52 mg/dL. Arterial blood gas analysis demonstrated a pH of 7.316, pCO₂ of 29.7 mmHg, pO₂ of 125 mmHg, HCO₃⁻ of 14.7 mmol/L, and lactate of 2.2 mmol/L (Table 1).

The patient was intubated, and CHDF was initiated for AKI on hospital day 1 owing to persistent oliguria and progression of metabolic acidosis; CHDF was discontinued on day 3. The patient was subsequently discharged from the ICU on hospital day 7.

## Discussion

In this report, we describe three cases of sepsis complicated by both AKI and EDKA managed in our ICU. EDKA accounts for approximately 2.6-3.2% of DKA cases, and although its prognosis remains uncertain, the diagnostic challenges associated with EDKA may contribute to a higher mortality rate than the reported 0.6-3% for conventional DKA [4]. Moreover, metabolic acidosis is common among critically ill patients, potentially obscuring the diagnosis of EDKA. Thus, the incidence of EDKA in the ICU may be higher than anticipated, underscoring the importance of accurate diagnosis and timely therapeutic intervention.

The etiologies of EDKA are intimately related to its underlying pathophysiology and are similar to those of DKA (Table 2).

**Table 2:** Cause of EDKA Causes of EDKA are listed in order of frequency. Although SGLT2 inhibitors are the most common precipitant, many ICU patients are exposed to additional risk factors such as infection, surgical invasiveness, and starvation, which likely predispose them to the development of EDKA. EDKA, euglycemic diabetic ketoacidosis; SGLT-2, sodium-glucose cotransporter-2

Cause	Frequency (%)
SGLT-2 inhibitor use	50
Starvation or prolonged fasting	15
Sepsis/infections	10
Pancreatitis	5
Cirrhosis/liver dysfunction	5
Alcohol use	4
Pregnancy	3
Trauma/surgery	3
Insulin deficiency or pump failure	2
Malignancy	2
Medications (steroids, thiazides)	1

In patients with type 2 diabetes, SGLT2 inhibitors are the most common precipitant of EDKA. An analysis of the Food and Drug Administration Adverse Reporting System indicated that approximately half to two-thirds of DKA cases may present with euglycemia [5]. Infection, acute physiological stress, starvation, and low-carbohydrate diets comprise the second most common group of causes, accounting for 10-20% of DKA cases [1, 6]. Infection and postoperative status stimulate the secretion of glucagon, cortisol, and catecholamines, thereby promoting insulin resistance, lipolysis, and ketogenesis despite normal blood glucose levels [7]. In addition, renal gluconeogenesis is impaired in AKI caused by sepsis, and hyperglycemia may be less likely to occur in cases with AKI caused by sepsis. All cases in our series were complicated by sepsis. Given that patients in the ICU are typically in a state of physiological stress, the incidence of EDKA may be underrecognized.

Metabolic acidosis is a common manifestation in both AKI and EDKA, and distinguishing between the two is challenging. In case 3, urinary ketones were found before ICU admission, leading to early suspicion of EDKA. However, in cases 1 and 2, the diagnosis of EDKA was not confirmed until blood ketones were measured. Rocktaeschel et al. analyzed acid-base disturbances in AKI patients using the Stewart approach and found that in AKI, the strong ion gap is elevated, with increased unmeasured anions such as ketone bodies and organic acids, while increases in chloride and phosphate levels were also observed [8]. This suggests that metabolic acidosis in AKI results not only from an increase in unmeasured anions containing non-volatile acids but also from factors such as phosphate and albumin. It is important to note that AKI can exist without an elevated anion gap, which can occur in the presence of lactate, unmeasured anions, ionized albumin, or phosphate. Therefore, AKI without an elevated anion gap is possible, but EDKA, characterized by the accumulation of unmeasured anions such as ketone bodies, will always result in an elevated anion gap. Although magnesium ion levels were not measured in our cases and the strong ion gap could not be analyzed, it is possible that measuring the strong ion gap could contribute to the diagnosis in cases in which differentiation between AKI and EDKA is difficult. On the other hand, a study of maintenance dialysis patients in the ICU reported a significant increase in sulfate, an unmeasured anion, which should be considered separately from AKI [9].

The diagnosis of EDKA is typically based on three criteria: relative euglycemia (<250 mg/dL), acidosis (pH <7.30, HCO₃⁻ <18 mEq/L), and ketosis (preferably serum beta-hydroxybutyrate >3 mmol/L, with serum acetoacetate or urine ketones as alternatives) [1]. The American College of Endocrinology also recommends the measurement of serum beta-hydroxybutyrate for screening EDKA [7]. However, the measurement of ketones, including serum beta-hydroxybutyrate, may take considerable time in many institutions. In our cases, although an elevated anion gap was present, lactate levels were within normal limits or only mildly elevated. Except for case 3, which exhibited septic shock, none of the patients were in overt shock; the combination of low lactate levels with an elevated anion gap metabolic acidosis prompted consideration of EDKA. In practice, in diabetic patients with risk factors for EDKA, as mentioned who meet the above criteria, the treatment for EDKA should be initiated while awaiting ketone results. Particularly in cases complicated by AKI, distinguishing between EDKA and AKI may be challenging, but given the limited indications for early kidney replacement therapy (KRT) in AKI, it seems prudent to prioritize EDKA treatment and consider KRT only if there is no improvement in metabolic acidosis during the treatment process. Although AKI often complicates DKA, treatment for DKA remains the priority in such cases, and the same applies to EDKA [10,11].

In all three cases, CHDF was initiated for AKI, which may have contributed to the early resolution of the metabolic acidosis associated with EDKA. Case reports on the efficacy of KRT in DKA, including EDKA, are limited. Although such therapies have been suggested to be beneficial in cases complicated by AKI [12], in patients on maintenance dialysis who develop EDKA, hemodialysis has not proven particularly effective [13]. Conversely, while KRT may offer benefits in DKA cases with concomitant AKI, its impact on overall prognosis remains uncertain and warrants further investigation.

## Conclusions

In summary, we reported three cases of sepsis complicated by both AKI and EDKA treated in our ICU. Although sepsis and significant physiological stress are risk factors for EDKA, diagnosing this condition in critically ill patients, especially in patients with AKI - who frequently present with metabolic acidosis - can be challenging. In diabetic patients with relevant risk factors, the presence of an elevated anion gap in the absence of elevated lactate levels should prompt consideration of EDKA; blood ketone levels should be measured and early insulin therapy initiated. The impact and efficacy of KRT on the prognosis of EDKA remains unclear, and KRT is not performed prior to the standard treatment for EDKA.
